# Theory of mind in juvenile myoclonic epilepsy

**DOI:** 10.1111/epi.70043

**Published:** 2025-12-04

**Authors:** Rafael Gustavo Sato Watanabe, Tatiana Goes Freitas, Emily Lima Marmentini, Maria Emilia Rodrigues de Oliveira Thais, Emil Kupek, Peter Wolf, Katia Lin

**Affiliations:** ^1^ Medical Sciences Graduate Program Federal University of Santa Catarina Florianópolis Brazil; ^2^ Neurology Division Federal University of Santa Catarina Florianópolis Brazil; ^3^ Medical Undergraduate Program Federal University of Santa Catarina Florianópolis Brazil; ^4^ Center for Applied Neurosciences Federal University of Santa Catarina Florianópolis Brazil

**Keywords:** cognition, executive function, neuropsychological tests, psychology

## Abstract

Juvenile myoclonic epilepsy (JME) is a common idiopathic generalized epilepsy often accompanied by executive dysfunction, affective symptoms, unfavorable behavior, and social outcomes, yet its impact on theory of mind (ToM) remains underexplored. We conducted an unmatched case–control study assessing 34 JME patients and 48 healthy controls, adjusted for age, sex, education, intelligence quotient, anxiety, and depression. Participants completed a brief version of the Faux Pas Recognition Test (FPRT) and the Reading the Mind in the Eyes Test, alongside measures of executive function, prospective memory, and mood. In raw analyses, JME patients showed significantly lower FPRT total scores (mean ± SD = 22.9 ± 9 vs. 27.5 ± 7, *p* = .01) and FPRT Understanding (.80 ± .11 vs. .87 ± .14, *p* = .02). After adjusting for cognitive and affective covariates via propensity scoring, group differences in ToM performance were no longer significant (*p* > .20). These results suggest that ToM deficits in JME are mediated by broader cognitive and affective disturbances rather than reflecting a social cognitive impairment. Future longitudinal studies with larger samples and tighter pharmacological control are warranted.

## 1 INTRODUCTION

Juvenile myoclonic epilepsy (JME) is a common idiopathic generalized epilepsy syndrome with onset in adolescence or early adulthood, clinically defined by myoclonic jerks, often on awakening, generalized myoclonic–tonic–clonic seizures, and variably present absences.^1^ Contemporary classification criteria from the International League Against Epilepsy (ILAE) require myoclonia and typical generalized spike/polyspike–wave (3–5.5 Hz) complexes on electroencephalogram (EEG), while specifying red flags and exclusionary features that guard against overdiagnosis. Clinical magnetic resonance imaging (MRI) is usually normal; abnormal findings prompt reconsideration of the diagnosis.^1^

Despite its longstanding reputation as a “benign” epilepsy, converging data show that many individuals with JME exhibit cognitive and behavioral vulnerabilities, particularly in executive function, attention, processing speed, and related frontal lobe skills.^2^ Psychiatric comorbidities, including anxiety, depression, and cluster B personality traits, are frequent.^3^ Social and functional outcomes can be suboptimal; a population study reported high rates of unplanned pregnancy, unemployment, and relational instability decades after onset.^4^ These broader phenotypic features have motivated research on underlying network abnormalities, subtle structural brain alterations, and putative genetic contributors to JME.^2^

Theory of mind (ToM), the capacity to attribute mental states (beliefs, intentions, emotions) to oneself and others, is fundamental to adaptive social behavior.^5^ Executive functions support ToM developmentally and may contribute to adult performance, although their role after maturation remains debated.^6^ Prospective memory (PM), another high‐level cognitive skill linked to goal management, may also be impaired in the JME population.^7^

Empirical data on ToM specifically in JME are scarce. Giorgi et al.^8^ reported mixed findings; selected Faux Pas Recognition Test (FPRT)^9^ scores and strange stories were impaired, but Reading the Mind in the Eyes Test (RMET)^10^ and portions of FPRT were not significantly different from controls; interpretation was limited by a small sample size and unclear selection criteria of FPRT stories for the short version. Morou et al.^11^ included a small JME subset within a broader generalized epilepsy group and found no robust ToM differences; sample heterogeneity again constrained inference. Preliminary functional imaging work with scant JME samples suggested greater psychiatric symptoms and poorer ToM, but definitive conclusions await larger, methodologically rigorous studies.^12^

People with epilepsy are exposed to multiple risk factors for cognitive impairment, including the underlying pathology, history of status epilepticus, antiseizure medication (ASM), and associated structural lesions. In JME, neuropsychiatric comorbidities, abnormalities on advanced neuroimaging, and unfavorable social outcomes may contribute to impairments in ToM. The few available studies specifically addressing ToM in JME are limited by methodological constraints, including small sample sizes and heterogeneous clinical profiles. These gaps highlight the need for further investigation to clarify ToM performance in JME and its potential associations with cognitive domains. Therefore, we conducted an unmatched case–control study comparing adults with JME to healthy controls adjusted by age, sex, schooling, intelligence quotient (IQ), and mood symptoms. Our primary hypothesis was that, relative to controls, individuals with JME would show poorer ToM performance; secondarily, we expected group differences in executive and PM measures and explored whether these, together with affective symptoms, accounted for ToM variability.

## 2 MATERIALS AND METHODS

We performed a cross‐sectional unmatched case–control study including structured interview, neuropsychological testing, and psychiatric symptom screening. All procedures adhered to the Declaration of Helsinki; written informed consent was obtained from all participants. The study was approved by the Federal University of Santa Catarina Human Research Ethics Committee. Data were collected between November 2022 and January 2025.

Eighty‐two adults (≥18 years old) participated: 34 with JME and 48 healthy controls recruited from hospital visitors/community contacts (nonrelatives of JME patients). Exclusion criteria included illiteracy, sensory impairments precluding testing, psychotropic drug use in controls, known progressive and uncontrolled psychiatric or neurologic diseases other than JME, and seizure occurrence within 24 h before assessment. All JME participants required normal cranial imaging (preferably MRI) and EEG consistent with ILAE JME criteria.

Structured interviews captured age, sex, schooling, marital/employment status, epilepsy history, and ASM use. Anxiety symptoms were screened with the Generalized Anxiety Disorder‐7 (GAD‐7); scores ≥ 10 indicated anxiety. Depression symptoms were assessed with the Neurological Disorders Depression Inventory for Epilepsy (NDDI‐E); scores > 15 suggested depression.

General intellectual ability (IQ estimate) was obtained with the Wechsler Abbreviated Scale of Intelligence (two‐subtest version). Executive functions were assessed with computerized Wisconsin Card Sorting Test (WCST), Stroop (Victoria version), Digit Span (Forward/Backward), and phonemic (F‐A‐S) and semantic (animals) fluency.

The Prospective Memory Task was performed where participants were instructed to perform two delayed actions (seal an addressed envelope and write their name on the back).^13^

For ToM evaluation, we used a Brazilian Portuguese version of the 36‐item RMET,^10^, ^14^ and a 10‐story Brazilian FPRT short version was constructed from psychometric data (discrimination indices) of the full Brazilian Portuguese adaptation (six faux pas, four control stories).^15^ Standard scoring produced domain scores: Detection, Understanding, Intention, Belief, Empathy, plus a faux pas total score (sum of first six questions across faux pas stories). Tests are detailed in the Supplementary Material.

### 2.1 Statistical analysis

Analyses were performed in IBM SPSS v26.0. For the primary objective (group differences in ToM), we estimated a propensity score via logistic regression including age, sex, schooling, IQ, GAD‐7, and NDDI‐E, covariates plausibly influencing ToM and differing between groups. Propensity scores were entered as covariates in logistic regression models comparing JME versus control status across ToM outcomes; multivariable models added neuropsychological variables after collinearity screening. Sociodemographic and neuropsychological variables were summarized as mean and SD. Group differences in raw scores were examined using the Mann–Whitney or independent‐samples *t*‐test, as appropriate. Statistically significant Spearman correlations, when present, were further explored using linear or logistic regression analyses.

Sample size was powered for a medium effect (*g* = .498) based on a meta‐analysis of ToM in generalized epilepsies, including a subset of JME patients,^16^ yielding a target of 34 individuals (*α* = .05, *β* = .20).

## 3 RESULTS

Mean age did not differ between groups (JME, 31.4 years; controls, 30.4 years); women comprised >60% in both. Schooling was lower in JME (12.3 years vs. 14.7 years, *p* = .001). Employment/education engagement was 58.8% in JME versus 100% in controls. IQ was lower in JME (97.2 vs. 118.4, *p* < .001). Anxiety (GAD‐7 ≥ 10) occurred in 61.8% of JME versus 20.9% of controls; depression (NDDI‐E > 15) occurred in 38.2% versus 8.3%, respectively (both *p* < .001; Table 1).

**TABLE 1:** Sociodemographic data and neuropsychological and ToM tests: Analysis of mean differences between JME and control groups.

	Control group, *n* = 48	JME group, *n* = 34	*p*
Sociodemographic			
Age, years	30.46 ± 12.74	31.38 ± 10.92	.733
Sex, female	60.4%	61.8%	.903
Schooling, years	14.73 ± 3.14	12.35 ± 3.26	.001
Marital status, married/stable relationship	20.8%	35.3%	.149
Profession, unemployed	0%	41.2%	.000
Neuropsychological			
GAD‐7	6.33 ± 4.12	11.32 ± 5.49	.000
NDDI‐E	10.46 ± 3.28	14.23 ± 4.91	.000
WASI vocabulary	65.62 ± 9.96	53.08 ± 14.06	.000
WASI matrix	28.04 ± 6.18	22.47 ± 8.53	.001
IQ	118.44 ± 19.92	97.17 ± 19.72	.000
Phonemic fluency–F	13.16 ± 3.92	9.23 ± 4.34	.000
Phonemic fluency–A	13.31 ± 3.65	8.73 ± 3.74	.000
Phonemic fluency–S	11.89 ± 3.94	8.73 ± 3.37	.000
Phonemic fluency–FAS	38.37 ± 10.18	26.70 ± 10.33	.000
Semantic fluency–animals	22.18 ± 5.25	15.82 ± 5.06	.000
Digit Span Forward	10.04 ± 2.45	7.94 ± 2.44	.000
Digit Span Backward	6.54 ± 2.34	5.11 ± 2.47	.010
Wisconsin	48.54 ± 9.00	39.52 ± 10.83	.000
Stroop–word reading	13.02 ± 2.77	18.38 ± 6.50	.000
Stroop–color naming	14.66 ± 4.45	21.02 ± 9.10	.000
Stroop–word/color interference	19.95 ± 8.43	31.38 ± 17.93	.000
Stroop correct answers	71.08 ± 1.54	69.82 ± 4.72	.088
Prospective letter	1.62 ± .70	1.14 ± .85	.007
Retrospective letter	.20 ± .50	.35 ± .69	.276
ToM			
RMET	26.43 ± 4.04	24.88 ± 4.32	.100
FP total	27.54 ± 7.00	22.91 ± 9.00	.011
FPRT Detection	.87 ± .11	.85 ± .13	.317
FPRT Understanding	.87 ± .11	.80 ± .14	.023
FPRT Intention	.75 ± .15	.70 ± .15	.137
FPRT Belief	.82 ± 0,12	.80 ± .13	.507
FPRT Empathy	.88 ± .10	.85 ± .13	.290

Abbreviations: FP, faux pas; FPRT, Faux Pas Recognition Test; GAD‐7, Generalized Anxiety Disorder‐7; IQ, intelligence quotient; JME, juvenile myoclonic epilepsy; NDDI‐E, Neurological Disorders Depression Inventory for Epilepsy; RMET, Reading the Mind in the Eyes Test; ToM, theory of mind; WASI, Wechsler Abbreviated Scale of Intelligence.

JME patients had an average of 18.9 years of epilepsy; the most used ASM was valproate (26.5%), and 47% used only one ASM; absence seizures were present in 35.3%, myoclonic–tonic–clonic seizures in 88.2%, and all presented myoclonic seizures. Nine individuals had at least weekly seizures, and seven individuals had fewer than one per year (JME profile detailed in Supplementary Material).

In JME patients, greater ASM use correlated with lower IQ (β = −.37, 95% confidence interval [CI] = −14.29 to −.88, *p* = .028, *R* ^2^ = .14), with an average decrease of 7.6 IQ points per additional drug. A similar negative trend was found for ToM (RMET; *B* = −1.43, β = −.324, *p* = .061). Working memory showed significant effects (Digit Span Backward; *B* = –1.03, β = −.407, *p* = .017, *R* ^2^ = .166) as did executive function (WCST; *B* = −5.18, β = −.469, *p* = .005, *R* ^2^ = .220), each additional ASM reducing performance by approximately one point and five correct responses, respectively. Stroop interference times also increased with higher ASM use (*B* = 7.35, β = .402, *p* = .018, *R* ^2^ = .162), reflecting slower processing and reduced inhibition. Seizure frequency and time of epilepsy were not correlated with neuropsychological tests.

Raw group comparisons showed broadly poorer executive performance in JME; phonemic and semantic fluency, Digit Span Forward/Backward, WCST, and longer Stroop completion times were seen across all conditions: word reading, color naming, and word/color interference (all *p* ≤ .01). PM (prospective envelope task) was reduced in JME (1.14 vs. 1.62, *p* = .007).

RMET scores trended lower in JME but were not statistically different (24.88 vs. 26.43, *p* = .100). On the FPRT, faux pas total and Understanding were lower in JME (faux pas total, 22.91 vs. 27.54, *p* = .011; Understanding, .80 vs. .87, *p* = .023). Detection, Intention, Belief, and Empathy subscores showed nonsignificant differences (Table 1).

When propensity scores (age, sex, schooling, IQ, anxiety, depression) were included, group effects on ToM measures attenuated and lost statistical significance. Similar nonsignificant odds ratios were observed across FPRT domains and RMET. Selected fluency tasks (letter A and animals) and Stroop–word reading were significantly worse in JME (all *p* ≤ .03; Table 2).

**TABLE 2:** Univariate and multivariate logistic regression analysis of neuropsychological and theory of mind tests (independent variables) among juvenile myoclonic epilepsy and control groups (dependent variables) adjusted by propensity score.

	Odds ratio	95% CI	*p*
Univariate analysis			
Phonemic fluency–F	.883	.760–1.024	.100
Phonemic fluency–A	.761	.639–.908	.002
Phonemic fluency–S	.828	.685–1.001	.051
Phonemic fluency–FAS	.943	.809–1.100	.457
Semantic fluency–animals	.825	.727–.935	.003
Digit Span Forward	.820	.643–1.045	.109
Digit Span Backward	.994	.772–1.280	.962
Wisconsin	.978	.919–1.040	.475
Stroop–word reading	1.263	1.052–1.516	.012
Stroop–color naming	1.070	.958–1.195	.229
Stroop–word/color interference	1.039	.977–1.104	.222
Stroop correct answers	.969	.779–1.206	.780
Prospective letter	.758	.365–1.576	.458
Retrospective letter	.786	.301–2.052	.623
RMET	1.034	.902–1.185	.635
FP total	.955	.887–1.028	.224
FPRT Detection	.201	.002–21.944	.503
FPRT Understanding	.072	.001–7.637	.269
FPRT Intention	.427	.010–18.528	.658
FPRT Belief	2.648	.031–223.865	.667
FPRT Empathy	.296	.002–37.031	.622
Multivariate analysis			
Age	.910	.803–1.032	.142
Sex	.334	.007–16.244	.580
Schooling	1.127	.772–1.645	.535
Marital status	.165	.009–3.106	.229
IQ	.902	.693–1.174	.442
GAD‐7	1.156	.767–1.745	.488
NDDI‐E	1.372	.593–3.174	.460
Phonemic fluency–A	.729	.542–.980	.036
Semantic fluency–animals	.822	.627–1.078	.156
Digit Span Forward	.740	.467–1.173	.200
Digit Span Backward	2.006	1.015–3.963	.045
Wisconsin	1.068	.928–1.229	.358
Stroop–word reading	1.535	1.037–2.271	.032
Prospective letter	1.355	.380–4.831	.639
RMET	.988	.759–1.286	.928
FPRT Detection	54.237	.005–652 102.589	.405

Abbreviations: CI, confidence interval; FP, faux pas; FPRT, Faux Pas Recognition Test; GAD‐7, Generalized Anxiety Disorder‐7; IQ, intelligence quotient; NDDI‐E, Neurological Disorders Depression Inventory for Epilepsy; RMET, Reading the Mind in the Eyes Test.

In a multivariate logistic regression analysis including sociodemographic, psychiatric, and neuropsychological variables after additional adjustment with exclusion due to high collinearity, only selected fluency (letter A) and Stroop (word reading) measures remained significant predictors of group status; ToM indices were not independently associated with JME after adjustment (Table 2).

## 4 DISCUSSION

In this study, adults with JME exhibited broad weaknesses in executive functions, lower IQ estimates, greater anxiety/depression symptom burden, and reduced prospective memory relative to healthy controls. However, ToM performance differences were modest and did not remain significant after adjusting for demographic, cognitive, and affective covariates. These data suggest that any apparent ToM decrement in JME may be secondary, mediated by executive inefficiencies, educational disparities, or mood/anxiety symptoms, rather than reflecting a primary deficit in ToM, as previously evidenced in the literature.^8^, ^11^, ^12^

Our findings expand on earlier small‐sample studies by incorporating propensity‐adjusted analyses, psychometrically validated measures, and a larger sample. Giorgi et al.^8^ reported selective ToM impairments but no RMET difference; interpretation was hampered by a short FPRT with unclear psychometrics and a sample (*n* = 20) underpowered to detect small‐to‐moderate effects. Morou et al.^11^ found no ToM differences in a heterogeneous generalized epilepsy cohort (JME, *n* = 9). A preliminary functional MRI series with seven JME participants suggested increased psychiatric symptoms and poorer ToM but lacked statistical power.^12^ By using a psychometrically supported Brazilian short FPRT (selected for discrimination indices) and controlling for key covariates via propensity modeling, we provide a more rigorous test of ToM in JME.

Our data show that adjusting for IQ, affective symptoms, and executive metrics substantially reduced JME group effects, aligning with the view that ToM task success in adult samples often depends on general cognitive resources.^17^ Moreover, PM, conceptually linked to intention formation, retention, and execution, was impaired in JME and could interfere with multistep story comprehension demands on the FPRT.^7^

The prevalence of anxiety and depression in our study was higher than in previous studies.^3^ Those along with personality traits have been repeatedly observed in JME and may influence social cognition through motivational, attentional, or interpretive biases.^5^ Population follow‐up underscores that psychosocial outcomes can remain challenging decades after seizure onset.^4^ Meta‐analytic work links depression and anxiety disorders to small‐to‐moderate ToM impairments, providing a plausible pathway by which mood symptoms could lower ToM test scores in epilepsy samples enriched for these comorbidities.^18^ Higher ASM load was associated with poorer performance across multiple cognitive domains, suggesting a potential pharmacological impact on cognition.^3^ This potential bias was partially mitigated by including depressive and anxiety scores in the propensity model, as these variables were positively correlated with both ASM use (ρ = .366, *p* = .033; ρ = .347, *p* = .044, respectively) and psychotropic medication use (ρ = .470, *p* = .005; ρ = .387, *p* = .024, respectively). The inclusion of patients under ASM and psychotropic medication reflects real‐world clinical conditions and enhances the ecological validity and clinical relevance of our findings.

No correlation was found between epilepsy duration and cognitive outcomes, including ToM. This may reflect cognitive consolidation in our sample, given the mean epilepsy duration of 18.9 years.

Clinically, modest ToM differences that dissipate after covariate adjustment argue against routine ToM screening in otherwise well‐functioning adults with JME. Instead, prioritizing management of executive dysfunction and mood/anxiety symptoms may yield broader benefits, including improved social participation.

### 4.1 Limitations

Cross‐sectional design precludes causal inference regarding cognitive mediators of ToM. Medication effects (type, load, serum levels) could not be fully controlled between groups, representing an inherent methodological limitation that may have influenced cognitive performance. Future work should be stratified by ASM exposure or assessed prior to treatment initiation. Although the study was adequately powered to detect medium effects, more subtle ToM cannot be ruled out, warranting replication with larger samples. Finally, community controls were more educated and fully employed or studying, without the use of psychotropic medication, leaving room for residual confounding. Nonetheless, these limitations would be expected to biased results toward worse ToM performance in JME, which was not observed, supporting the robustness of our findings.

## 5 CONCLUSIONS

Adults with JME show broad executive, PM, and affective vulnerabilities but no robust, independent impairment in ToM when these factors are considered. Social–cognitive complaints in JME may therefore reflect downstream effects of generalized cognitive load and mood disturbance rather than a syndrome‐specific ToM deficit. Longitudinal, multicentric studies integrating neuroimaging, genetic, and ecological social functioning metrics are needed to delineate developmental trajectories and treatment targets.

## AUTHOR CONTRIBUTIONS

*Study design, screening for patients, and writing—review and editing:* Rafael Gustavo Sato Watanabe, Tatiana Goes Freitas, Emily Lima Marmentini, Maria Emilia Rodrigues de Oliveira Thais, Emil Kupek, Peter Wolf, and Katia Lin. *Data analyses:* Rafael Gustavo Sato Watanabe and Emil Kupek. Writing—original draft: Rafael Gustavo Sato Watanabe.

## CONFLICT OF INTEREST STATEMENT

K.L. holds a National Council for Scientific and Technological Development–CNPq PQ1C Research Fellowship (process number 306916/2023–1). The other authors do not have any conflict of interest to disclose. We confirm that we have read the Journal's position on issues involved in ethical publication and affirm that this report is consistent with those guidelines.

## Supporting information


Data S1.

Table S1.

## Data Availability

The data that support the findings of this study are available from the corresponding author upon reasonable request.
