# Supplementary material for: Theory of mind in juvenile myoclonic epilepsy
Source: Epilepsia. 2025 Dec 4;67(1):e1–7. doi: 10.1111/epi.70043 (PMC12893254; doi:10.1111/epi.70043)
Supplement: Supplementary file 1 — Data S1. [file EPI-67-e1-s002.docx]

**Supplementary Methods**

**Faux Pas Recognition Test**

The Faux Pas Recognition Test (FPRT) was developed by Stone et al. (1998)^1^ and was based on a children’s version designed by Baron-Cohen et al. (1999)^2^ for the assessment of Theory of Mind (ToM). The term *faux pas*, of French origin, literally means “false step” and, in the context of this test, refers to a socially awkward situation resulting from a verbal blunder, as illustrated in the test stories.

The original instrument consists of 10 *faux pas* (FP) stories (numbers 2, 4, 7, 11, 12, 13, 14, 15, 16, 18) and 10 control stories (numbers 1, 3, 5, 6, 8, 9, 10, 17, 19, 20), presented in random order. After each story is read aloud by the examiner—while the participant has a printed copy available—a sequence of questions is administered. The first two questions assess *faux pas* detection: (1) Did someone say something they should not have, or something inappropriate? If yes: (2) Who said it? The third question assesses understanding of inappropriateness: (3) Why should he/she not have said that, or why was it inappropriate? The fourth question evaluates intention—the motives or intentions of the character: (4) Why do you think he/she said that? The fifth question assesses belief—a true or false belief held by the character: (5) Did X know/realize that Y…? The sixth question measures empathy: (6) How do you think X felt? The seventh and eighth control questions assess comprehension of the story.

**Faux Pas Recognition Test – Short Version**

Based on a previous publication^3^ that evaluated the psychometric properties of a Brazilian version, 10 stories were selected. Watanabe et al. (2021)^3^ concluded that the control stories did not correlate with the rest of the test and showed low discriminative indices. Therefore, four control stories and six *faux pas* stories were included in the short version. The discriminative index guided the selection of the most sensitive stories for the abbreviated format. The most discriminative FP stories were numbers 16, 13, 7, 18, and 12. FP stories 2, 14, and 15 had identical discriminative indices (0.45); story 2 was chosen based on contextual relevance and expert consensus from the Federal University of Santa Catarina (UFSC) Neurology Group. Using the same descending discriminative criterion, four control stories were selected (1, 9, 20, 5). Among FP stories, numbers 4 and 11 presented the lowest discriminative indices; story 4 was retained by consensus among experts from the UFSC Neurology Group as an illustrative example. Stories were presented in the following sequence: 16, 5, 2, 12, 1, 13, 9, 18, 7, 20, ensuring a balanced distribution between FP and control stories.

**Faux Pas Recognition Test – Scoring and Scores**

According to the scoring procedures described by Stone and Baron-Cohen (2016)^4^, five subscores were computed independently (FP Detection, Understanding of Inappropriateness, Intention, Belief, and Empathy). Following Söderstrand and Almkvist (2012)^5^, a Total FP Score was also calculated. Participants received 1 point for each correct response among the eight questions per story. The scores for each question of each FP and control story were summed to produce the subscores (FP Detection – questions 1–2; Understanding of Inappropriateness – question 3; Intention – question 4; Belief – question 5; Empathy – question 6). Control questions (7–8) were used to verify comprehension. The FP Detection score ranged from 0 to 1. The subscores for Understanding of Inappropriateness, Intention, Belief, and Empathy ranged from 0 to 0.5 and were doubled to match the 0–1 range. The Total FP Score ranged from 0 (no correct responses) to 36 points (all six FP story questions answered correctly).

**Reading the Mind in the Eyes Test (RMET)**

The RMET assesses the individual’s ability to infer others’ intentions, feelings, and thoughts from facial expressions—specifically, the eye region. The test was first described by Baron-Cohen et al. (2001)^6^, who demonstrated that contextual information alone is insufficient to determine another person’s intentions. They proposed that people rely on facial cues to infer others’ emotions and desires, and that these cues are mentally represented according to individual cognitive characteristics, corresponding to inferred mental states.

The RMET can be administered using either a paper-and-pencil or a computerized version. It is simple to apply and does not require specialized training. The revised original version consists of 36 black-and-white photographs depicting the same facial area (from mid-nose to just above the eyebrows) and includes one practice item. Each image is accompanied by four mental-state descriptors, and participants must choose the one that best matches the expression. The test includes a glossary of all words used, with synonyms and example sentences, which should be reviewed before administration. The RMET is generally scored as a total score.

The Brazilian Portuguese version was translated and validated by Sanvicente-Vieira et al. (2013)^7^ and reflects the affective component of ToM^8,9^.

**Generalized Anxiety Disorder 7 (GAD-7)**

The Generalized Anxiety Disorder 7 (GAD-7) is a brief screening instrument designed for the assessment, diagnosis, and monitoring of anxiety symptoms. Developed by Spitzer et al. (2006)^10^ and validated by Kroenke et al. (2007)^11^ according to DSM-IV criteria, it evaluates the frequency of anxiety-related signs and symptoms over a two-week period. The scale comprises seven items rated on a 4-point Likert scale, ranging from 0 (“not at all”) to 3 (“nearly every day”), with total scores ranging from 0 to 21. Scores ≥10 were considered indicative of clinically relevant anxiety symptoms. Severity was classified as follows: 0–4 (minimal anxiety), 5–9 (mild), 10–14 (moderate), and 15–21 (severe anxiety).

**Neurological Disorders Depression Inventory for Epilepsy (NDDI-E)**

The Neurological Disorders Depression Inventory for Epilepsy (NDDI-E) is a brief, six-item instrument designed for rapid screening (approximately three minutes) of depressive episodes in individuals with epilepsy. Developed by Gilliam et al. (2006)^12^ and translated/validated into Brazilian Portuguese by Oliveira et al. (2010)^13^, it considers scores >15 indicative of depression. Although originally developed for individuals with epilepsy—minimizing overlap with antiepileptic drug side effects or cognitive comorbidities—it may also be applied to healthy control participants as in this study.

**Intelligence**

The Wechsler Abbreviated Scale of Intelligence (WASI) is a brief measure of general intelligence, with an average administration time of 30–45 minutes. It comprises four subtests: two Verbal (Vocabulary, Similarities) and two Performance (Block Design, Matrix Reasoning), and is suitable for individuals aged 6 to 89 years. The WASI provides three composite IQ measures: Full Scale, Verbal, and Performance IQ. For time efficiency, only the Vocabulary and Matrix Reasoning subtests were administered to estimate Full-Scale IQ^14,15^.

**Executive Function**

Executive function was assessed using the Wisconsin Card Sorting Test (WCST), Stroop Test, Digit Span (forward and backward), and verbal fluency tasks (semantic and phonemic). The Wisconsin Card Sorting Test (WCST)^16^ evaluates cognitive flexibility and the ability to shift problem-solving strategies. The computerized version presents cards varying in shape, color, and number of symbols. Participants must infer the categorization rule and adapt when the rule changes without warning. The computerized and traditional card-based versions are equivalent in psychometric performance^17^.

Inhibitory control and selective attention were assessed using the Stroop Test^18^, which measures the ability to inhibit an automatic reading response in favor of naming the color of ink in incongruent word–color pairs (e.g., the word “red” printed in blue). The Victoria version consists of three conditions: (1) naming the color of solid blocks, (2) naming the color of printed words, and (3) naming the color of incongruent color words. The test engages several cognitive mechanisms, including processing speed, working memory, semantic activation, cognitive flexibility, and inhibitory control^19^. Performance on the Stroop Test was quantified as completion time (in seconds) for each card. The interference score was computed as the difference between color-word and color-naming conditions. Higher scores reflect greater susceptibility to interference.

The Digit Span Test^20^ assesses short-term verbal memory, working memory, and attention. In the forward condition, the examiner reads a sequence of digits that the participant repeats in the same order; in the backward condition, the participant repeats the digits in reverse order, with increasing sequence length.

Verbal fluency tasks evaluate the ability to generate words under timed constraints. Two conditions were used: phonemic fluency (producing as many words as possible beginning with F, A, and S within one minute^21^; and semantic fluency (producing as many animal names as possible within one minute)^22^.

**Prospective Memory**

Based on Huppert et al. (2000)^23^, a task assessing prospective memory was administered. After participants labeled an envelope, they were instructed: “Later, I will ask you to write a name and address on this envelope. When you finish, please seal it and write your own name on the back. Can you remember to do that without me reminding you?”

After approximately 10 minutes—during which other cognitive tasks (fluency and Digit Span) were administered—the envelope was presented again, and the examiner said: “Please write the following name and address on this envelope: Fernando Silva, Rua Lauro Linhares, 58 – Trindade – Florianópolis.” The researcher dictated the information slowly, pausing after each component.

The examiner observed whether the participant carried out the instructed prospective memory task. If no action was taken within 5–10 seconds (longer for slower participants), the examiner prompted: “Should you be doing something with the envelope?” If only one correct action was performed (e.g., sealing or writing the name on the back), the examiner asked: “Is there something else you should do?” Prospective memory responses were scored as 2, 1, or 0 points depending on the number of correct actions performed independently. For participants who initially scored 0, an additional score (2, 1, or 0) was given for correct responses after the prompt, representing the retrospective memory component.

**References**

1. Stone VE, Baron-Cohen S, Knight RT. Frontal lobe contributions to theory of mind. *J Cogn Neurosci*. 1998 Sep;10(5):640-656. doi:10.1162/089892998562942.
2. Baron-Cohen S, Wheelwright S. The Empathy Quotient: an investigation of adults with asperger syndrome or high functioning autism, and normal sex differences. *J Autism Dev Disord*. 2004 Apr;34(2):163-175. doi:10.1023/b:jadd.0000022607.19833.00.
3. Watanabe RGS, Knochenhauer AE, Fabrin MA, Siqueira HH, Martins HF, Mello CDO, Zingano BL, Botelho MF, Yacubian EMT, Oliveira Filho GR, Melo HM, Walz R, Wolf P, Lin K. Faux Pas Recognition Test: Transcultural adaptation and evaluation of its psychometric properties in Brazil. *Cogn Neuropsychiatry*. 2021 Jun 16;1–14. doi:10.1080/13546805.2021.1941830.
4. Stone V, Baron-Cohen S. *Faux Pas Recognition Test (adult version)* [Internet]. 2016 [cited 2016 Mar 1]. Available from: http://docs.autismresearchcentre.com/tests/FauxPas_Adult.pdf
5. Söderstrand P, Almkvist O. Psychometric data on the Eyes Test, the Faux Pas Test, and the Dewey Social Stories Test in a population-based Swedish adult sample. *Nordic Psychology*. 2012 Mar;64(1):30–43. doi:10.1080/19012276.2012.693729.
6. Baron-Cohen S, Wheelwright S, Hill J, Raste Y, Plumb I. The “Reading the Mind in the Eyes” Test revised version: A study with normal adults, and adults with Asperger syndrome or high-functioning autism. *J Child Psychol Psychiatry*. 2001 Feb;42(2):241–51. doi:10.1111/1469-7610.00715.
7. Sanvicente-Vieira B, Kluwe-Schiavon B, Wearick-Silva LE, Piccoli GL, Scherer L, Tonelli HA, Grassi-Oliveira R. Revised Reading the Mind in the Eyes Test (RMET) – Brazilian version. *Rev Bras Psiquiatr*. 2014 Jan-Mar;36(1):60–7. doi:10.1590/1516-4446-2013-1162.
8. Shamay-Tsoory SG, Shur S, Barcai-Goodman L, Medlovich S, Harari H, Levkovitz Y. Dissociation of cognitive from affective components of theory of mind in schizophrenia. *Psychiatry Res*. 2007 Jan;149(1–3):11–23. doi:10.1016/j.psychres.2005.10.018.
9. Duval C, Piolino P, Bejanin A, Eustache F, Desgranges B. Age effects on different components of theory of mind. *Conscious Cogn*. 2011 Sep;20(3):627–42. doi:10.1016/j.concog.2010.10.025.
10. Spitzer RL, Kroenke K, Williams JB, Löwe B. A brief measure for assessing generalized anxiety disorder. *Arch Intern Med*. 2006 May 22;166(10):1092. doi:10.1001/archinte.166.10.1092.
11. Kroenke K, Spitzer RL, Williams JB, Monahan PO, Löwe B. Anxiety disorders in primary care: prevalence, impairment, comorbidity, and detection. *Ann Intern Med*. 2007 Mar 6;146(5):317–25. doi:10.7326/0003-4819-146-5-200703060-00004.
12. Gilliam FG, Barry JJ, Hermann BP, Meador KJ, Vahle V, Kanner AM. Rapid detection of major depression in epilepsy: a multicentre study. *Lancet Neurol*. 2006 May;5(5):399–405. doi:10.1016/S1474-4422(06)70415-X.
13. Oliveira GNM, Kummer A, Salgado JV, Portela EJ, Sousa-Pereira SR, David AS, Kanner AM, Teixeira AL. Brazilian version of the Neurological Disorders Depression Inventory for Epilepsy (NDDI-E). *Epilepsy Behav*. 2010 Nov;19(3):328–31. doi:10.1016/j.yebeh.2010.07.013.
14. Heck VS, Yates DB, Poggere LC, Tosi SD, Bandeira DR, Trentini CM. Validação dos subtestes verbais da versão de adaptação da WASI. *Aval Psicol*. 2009;8(1):33–42.
15. Yates DB, Trentini CM, Tosi SD, Corrêa SK, Poggere LC, Valli F. Apresentação da Escala de Inteligência Wechsler Abreviada (WASI). *Aval Psicol*. 2006;5(2):227–33.
16. Heaton RK, Chelune GJ, Talley JL, Kay GG, Curtiss G. *Teste Wisconsin de Classificação de Cartas*. São Paulo: Casa do Psicólogo; 2004. 346 p.
17. Wagner GP, Trentini CM. Assessing executive functions in older adults: a comparison between the manual and the computer-based versions of the Wisconsin Card Sorting Test. *Psychol Neurosci*. 2009 Jul;2(2):195–8. doi:10.3922/j.psns.2009.2.011.
18. Stroop JR. Studies of interference in serial verbal reactions. *J Exp Psychol*. 1935 Dec;18(6):643–62. doi:10.1037/h0054651.
19. Strauss E, Sherman EMS, Spreen O. *A compendium of neuropsychological tests: administration, norms, and commentary*. 3rd ed. New York: Oxford University Press; 2006.
20. Wechsler D. *WAIS-III: Escala de Inteligência Wechsler para Adultos: manual técnico*. São Paulo: Casa do Psicólogo; 2004.
21. Machado TH, Fichman HC, Santos EL, Carvalho VA, Fialho PP, Koenig AM, Fernandes CS, Lourenço RA, Paradela EMP, Caramelli P. Normative data for healthy elderly on the phonemic verbal fluency task – FAS. *Dement Neuropsychol*. 2009 Mar;3(1):55–60. doi:10.1590/S1980-57642009DN30100011.
22. Brucki SMD, Malheiros SMF, Okamoto IH, Bertolucci PHF. Dados normativos para o teste de fluência verbal categoria animais em nosso meio. *Arq Neuropsiquiatr*. 1997;55(1):56–61. doi:10.1590/S0004-282X1997000100009.
23. Huppert FA, Johnson T, Nickson J. High prevalence of prospective memory impairment in the elderly and in early-stage dementia: Findings from a population-based study. *Appl Cogn Psychol*. 2000 Jan;14(7):S63–81. doi:10.1002/acp.771.
