# Supplementary material for: Theory of mind in juvenile myoclonic epilepsy
Source: Epilepsia. 2025 Dec 4;67(1):e1–7. doi: 10.1111/epi.70043 (PMC12893254; doi:10.1111/epi.70043)
Supplement: Supplementary file 2 — Table S1. [file EPI-67-e1-s001.docx]

**Table - JME clinical profile**

| **Patient** | **Age** | **Sex** | **Antiseizure medication*** | **Psychotropic medication*** | **Years of epilepsy** | **Seizures/month** | **Seizure type** |
| --- | --- | --- | --- | --- | --- | --- | --- |
| **1** | 25 | M | VPA 1000mg | - | 8 | 1.0 | MYO |
| **2** | 20 | M | VPA 750mg | - | 17 | 4.0 | MYO, ABS |
| **3** | 31 | F | TPM 200mg, VPA 800mg, LEV 2000mg | ESC 10mg | 11 | 1.0 | MYO, ABS |
| **4** | 33 | F | VPA 500mg | - | 15 | 0.017 | MYO, MTCS |
| **5** | 44 | F | VPA 1000mg, LEV 1000mg | QUET 100mg, SER 50mg, DUL 60mg | 38 | 1.0 | MYO, MTCS, ABS |
| **6** | 45 | F | - | - | 34 | 0.17 | MYO, MTCS |
| **7** | 45 | F | LTG 200mg, CZP 4mg, LCM 400mg | VNF 150mg, QUET 25mg | 31 | 0.25 | MYO, MTCS |
| **8** | 21 | M | VPA 2000mg, CZP 1,5mg | - | 15 | 0.25 | MYO, MTCS |
| **9** | 58 | M | PB 100mg, CBZ 600mg, VPA 1500mg | - | 29 | 0.05 | MYO, MTCS |
| **10** | 27 | F | VPA 500mg, LEV 250mg | - | 9 | 30.0 | MYO, MTCS |
| **11** | 18 | M | VPA 1250mg | - | 4 | 0.33 | MYO, MTCS |
| **12** | 26 | F | LEV 1000mg | - | 10 | 1.0 | MYO, MTCS |
| **13** | 38 | F | LEV 1250mg | - | 22 | 0.5 | MYO, MTCS |
| **14** | 26 | M | VPA 1250mg, LEV 1500mg, LTG 100mg, CZP 1mg | - | 22 | 4.0 | MYO |
| **15** | 35 | M | VPA 1500mg, PB 100mg | - | 22 | 1.0 | MYO, MTCS, ABS |
| **16** | 29 | M | - | - | 15 | 1.5 | MYO, MTCS, ABS |
| **17** | 33 | F | VPA 2000mg, LEV 2250mg | CPZ 15mg, RIS 1mg | 24 | 2.0 | MYO, MTCS |
| **18** | 31 | F | LEV1500mg, VPA 1000mg | IMI 25mg | 19 | 30.0 | MYO, MTCS, ABS |
| **19** | 37 | F | LTG 100mg | - | 22 | 0.05 | MYO, MTCS, ABS |
| **20** | 25 | F | VPA 2000mg | SER 200mg | 18 | 4.0 | MYO, MTCS, ABS |
| **21** | 18 | M | VPA 1000mg | - | 12 | 0.33 | MYO, MTCS |
| **22** | 39 | M | PB 100mg | - | 31 | 0.014 | MYO, MTCS |
| **23** | 58 | F | LEV 750mg | - | 50 | 0.05 | MYO, MTCS |
| **24** | 18 | M | LEV1500mg | - | 12 | 0.05 | MYO, MTCS |
| **25** | 19 | F | VPA 2000mg | - | 8 | 0.75 | MYO, MTCS |
| **26** | 36 | F | LTG 350mg, PB 100mg | SER 50mg | 25 | 1.0 | MYO, MTCS |
| **27** | 20 | F | LTG 200mg | SER 100mg | 0 | 30.0 | MYO, MTCS |
| **28** | 18 | M | VPA 1000mg | - | 2 | 0.05 | MYO, MTCS, ABS |
| **29** | 41 | F | LEV 1500mg, LTG 300mg | - | 25 | 0.5 | MYO, MTCS |
| **30** | 29 | F | LEV 3000mg, LTG 500mg, PB 200mg | LIT 900mg | 11 | 4.0 | MYO, MTCS, ABS |
| **31** | 23 | F | LTG 400mg, CZP 4mg | SER 50mg | 10 | 2.0 | MYO, MTCS |
| **32** | 46 | F | LEV 2500, LTG 200mg, CLB 20mg, VPA 750mg | - | 42 | 4.0 | MYO, MTCS, ABS |
| **33** | 26 | F | VPA 500mg | ESC 15mg | 2 | 30.0 | MYO, MTCS |
| **34** | 29 | M | VPA 1000mg | - | 9 | 0.42 | MYO, MTCS, ABS |

*medications daily dosage; ABS: absence seizure; CLB: clobazam; CZP: clonazepam; CPZ: chlorpromazine; DUL: duloxetine; ESC: escitalopram; F:female; IMI: imipramine; LCM: lacosamide; LEV: levetiracetam; LIT: lithium; LTG: lamotrigine; M: male; MTCS: myoclonic-tonic-clonic seizure; MYO: myoclonic seizure; PB: phenobarbital; QUET: Quetiapine; RIS: risperidone; SER: sertraline; VPA: valproic acid; VNF: Venlafaxine
